# Mechanochemical synthesis of carbon-stabilized Cu/C, Co/C and Ni/C nanocomposites with prolonged resistance to oxidation

**DOI:** 10.1038/s41598-019-54007-2

**Published:** 2019-11-22

**Authors:** Mariia Galaburda, Evgeniya Kovalska, Benjamin T. Hogan, Anna Baldycheva, Andrii Nikolenko, Galina I. Dovbeshko, Olena I. Oranska, Viktor M. Bogatyrov

**Affiliations:** 1 Oxide Nanocomposites Laboratory, Chuiko Institute of Surface Chemistry of NAS of Ukraine, 17 General Naumov Str, Kyiv, 03164 Ukraine; 2 Department of Engineering and Centre for Graphene Science, College of Engineering, Mathematics and Physical Sciences, University of Exeter, Exeter, EX4 4QF United Kingdom; 3 Optical Submicron Spectroscopy Laboratory, Institute of Semiconductor Physics of NAS of Ukraine, 45 Nauky Ave, Kyiv, 02000 Ukraine; 4 Department of Physics of Biological Systems, Institute of Physics of NAS of Ukraine, 46 Nauky Ave., Kyiv, 02000 Ukraine

**Keywords:** Chemistry, Materials science, Nanoscience and technology

## Abstract

Metal-carbon nanocomposites possess attractive physical-chemical properties compared to their macroscopic counterparts. They are important and unique nanosystems with applications including in the future development of nanomaterial enabled sensors, polymer fillers for electromagnetic radiation shields, and catalysts for various chemical reactions. However, synthesis of these nanocomposites typically employs toxic solvents and hazardous precursors, leading to environmental and health concerns. Together with the complexity of the synthetic processes involved, it is clear that a new synthesis route is required. Herein, Cu/C, Ni/C and Co/C nanocomposites were synthesized using a two-step method including mechanochemical treatment of polyethylene glycol and acetates of copper, nickel and cobalt, followed by pyrolysis of the mixtures in an argon flow at 700 °C. Morphological and structural analysis of the synthesized nanocomposites show their core-shell nature with average crystallite sizes of 50 (Cu/C), 18 (Co/C) and 20 nm (Ni/C) respectively. The carbon shell originates from disordered sp^2^ carbon (5.2–17.2 wt.%) with a low graphitization degree. The stability and prolonged resistance of composites to oxidation in air arise from the complete embedding of the metal core into the carbon shell together with the presence of surface oxide layer of metal nanoparticles. This approach demonstrates an environmentally friendly method of mechanochemistry for controllable synthesis of metal-carbon nanocomposites.

## Introduction

Metal nanoparticles are attracting considerable attention due to their huge potential in developing a broad range of efficient technologies. However, the use of pure metal nanoparticles is difficult owing to their instability owing to oxidation in air, agglomeration, dissolution in acid, and others. This limits their potential use in both industry and scientific research. To overcome these limitations, the application of a protective shell has been recommended as a prospective method to improve the chemical stability of metal nanoparticles. Various protective coating materials have been proposed, *e.g*. polymer silica, carbon *etc*.^1–4^. Due to its high stability both chemically and physically, good biocompatibility and high surface activity, carbon is considered the most desirable material for encapsulation. A carbon shell around metal-containing nanocomposites can ensure the preservation of the size and physicochemical properties of metastable nanocrystalline materials for a long time. Additionally, the carbon coating itself has unique structural, adsorption, electronic, and mechanical characteristics. Owing to the diversity of their structural forms and peculiar properties, carbon nanostructures have been gaining ground in materials science and enabling various applications^5^.

Carbon nanocomposite materials with metal nanoparticles such as Cu, Co, or Ni or their oxides are interesting for a broad range of applications - from magnetic resonance tomography and biomedicine, to electrode materials of supercapacitors, and heterogeneous catalysts of petrochemical processes, to components of disperse radiation absorbing media^6–10^. Metal-carbon nanocomposites- possessing very promising physical, chemical and mechanical properties - are of special interest to researchers due to their broad applicability as heterogeneous catalysts for different chemical reactions. Different metal-carbon containing catalytic systems exhibit high activity in various reactions, such as hydrogenation, isomerization, and cross-coupling^11–14^. Additionally, the biocidal properties of copper nanopowders are used in medicine to create anti-bacterial materials^15^. Furthermore, metallic conductive fillers are widely used to impart specific properties to polymer composite materials. Powders of iron, aluminum, copper, zinc, silver, along with alloys of cobalt, nickel, manganese and iron are used for these purposes. Metal powders can have various shapes - spherical, scaly, and dendritic. The size, shape and nature of the surface of metal particles have a major impact on the properties of the polymer composites^16^. Owing to their high electrical conductivity, metal/carbon nanocomposites (*e.g*. Co/C) have been used to create electrically conductive polymeric materials and electromagnetic radiation shields^17^. Cu/C nanocomposites, due to their good thermal conductivity and low friction coefficient, have been employed for seam welding machine applications, where the graphite creates the secondary phase in the matrix thus ensuring high sliding properties^18^. Ni/C nanocomposites, as an important member of the family of carbon-encapsulated metal nanoparticles, have received much attention due to their exceptional electromagnetic wave absorption properties^19^. It is worth noting that metal-carbon composites have proven themselves as suitable magneto-sensitive sorbents for the adsorption of explosives, water pollutants and dyes^20–22^. The sorbents possess high adsorption capacity and advanced magnetic characteristics, and therefore can be quickly and efficiently separated from the solutions using magnetic field, or they can be brought by the magnet to the required nodes of the equipment for cleaning.

Different approaches to obtain carbon-protected metal nanoparticles with a metal core and a graphitic shell have been reported^23–25^. It is known that organic reactions, especially at large scales, are routinely carried out in a solution phase. Unfortunately, most organic solvents used for this purpose are volatile organic compounds and these cause serious environmental problems. The proposed mechanochemical method is generally accepted waste-free and ecologically safe method, which involves the mechanical milling of solid-state materials/chemicals and has been widely used in synthesise of the metallic and oxide nanoparticles, catalysts, and biological materials^27,28^. The mechanochemical process makes it possible to choose safe, non-toxic materials, applying renewable materials, as well as to reduce the energy consumption and the return of harmful substances to the environment. Additionally, the polyethylene glycol polymer proposed for use herein as the source of carbon structures is inexpensive and significantly less hazardous than other polymers^26^. Hence, mechanochemistry is a promising direction of solid chemistry to reach highly sought-after Green Chemistry goals^29^.

With this in mind, the presented work describes a mechanical treatment as a non-conventional solid-state process for the preparation of metal-doped polymer composites for further there pyrolysis and formation of new functional carbon nanomaterials. Aiming for a better understanding of the structure/property relationship and the development of novel functional materials, a complex study of the morphology and structure of Cu-, Co- and Ni-carbon nanocomposites was conducted, using modern analytic methods such as scanning electron microscopy, transmission electron microscopy and Raman spectroscopy.

## Results and Discussion

As part of the line of research focussing on the synthesis and study of the structural properties of carbon-inorganic nanocomposites, this work is devoted to the study of the structure of Cu-, Co- and Ni-containing carbon composites with high metal content ( > 80 wt.%). The nanocomposites were synthesised as described in the Materials and Methods section. The pyrolyzed solid materials obtained were black voluminous powders. The possibility of the formation of a graphitic form in the metal-carbon nanocomposites obtained from polymers of a different nature has been shown elsewhere^30^.

The compositions of the products after carbonisation were determined by X-ray diffraction (XRD) (see Supplementary Fig. S1 and Table S2). The XRD patterns confirm that Ni, Co, and Cu were completely reduced to the metallic state after pyrolysis. The patterns of the samples reveal the presence of face-centered cubic crystalline structures of nickel (JCPDS No. 4–850), copper (JCPDS No. 4–856), and cobalt (JCPDS No. 15–806) with traces of hexagonal cobalt (JCPDS No. 5-727). There is a reflex related to the structure of graphite, with diffraction indices (002) on the XRD patterns of the Ni/C and Co/C samples. The relatively low intensity of the carbon (002) diffraction peak for the Ni/C and Co/C composites and its absence for the Cu/C could be related to the disordered nature of the graphite-like sp^2^ carbon and to the low weight content of carbon in the Cu/C composite (about 5.6 wt.%), correspondingly. The average crystallite size of the metals was 18–20 nm for Co/C and Ni/C nanocomposites respectively, and 50 nm for Cu/C sample (Fig. S1, Table S1). The dimension of the nanographite in the as-prepared samples was 5–7 nm (Table S1). We should note here that no carbide phases were found in any of the samples. The Cu nanoparticles are approximately twice the size of the Ni and Co. Such correlation of the average sizes of the nanocomposites is connected with the ability to form metal containing clusters. Thus, the synthesised nanocomposites demonstrate magnetic properties that definitely widens their application^31^.

The specific surface area of powders of the Ni/C, Co/C, and Cu/C nanocomposites were calculate to be 28, 94, and 29 m^2^/g, respectively. The obtained results confirm the presence of mesopores in the nanocomposites with the total pore volume of 43 (Ni/C), 42 (Co/C), and 36 mm³/g **(**Cu/C) (see Supplementary Fig. S2 and Table S2). The ratio of metal and carbon in the nanocomposites was determined based on thermogravimetry analysis and the phase composition of the initial and heated samples after derivatography. Detailed descriptions of the calculations are given in the previous work^32^. The metal content in the composites was 94.4, 84.8, 82.8 wt.%, while carbon – 5.6, 15.2 and 17.2 wt.% for Cu/C, Ni/C and Co/C composites, respectively.

**Figure 1:**
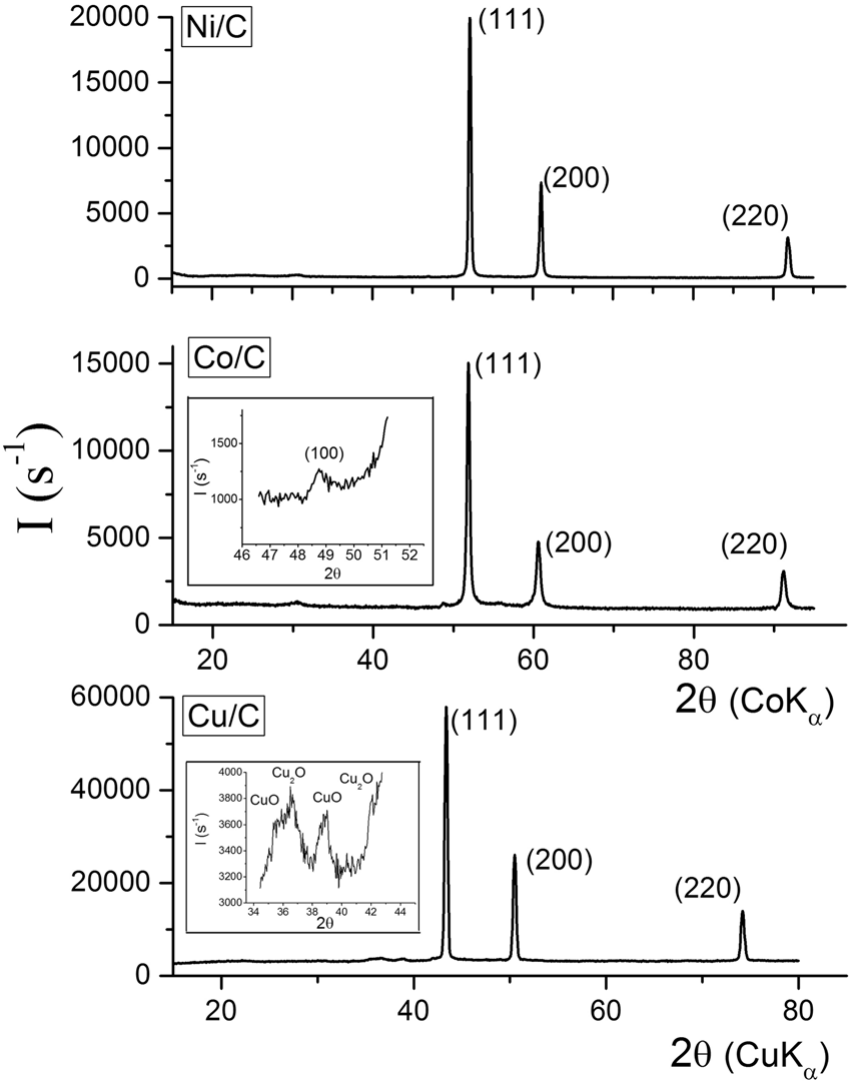
XRD patterns of Ni/C, Co/C, and Cu/C nanocomposites obtained via pyrolysis under argon flow at 700 °C after three years of storage *under an air atmosphere*, recorded using diffractometers with filtered CuK_α_ radiation for Cu/C, and monochromatic CoK_α_ radiation for Co/C and Ni/C nanocomposites. The diffraction patterns show peaks related to the (111), (200) and (220) planes of face-centered cubic crystalline structures of metallic Ni, Co, and Cu with a trace amount of hexagonal cobalt (100) and copper oxides CuO, Cu_2_O (in inserts).

It is worth noting that the as-prepared composites have kept their aggregate state, remained loose and dispersed after three years of exposure to an air atmosphere. Therefore, the samples were further studied by the XRD method (Fig. 1). The diffraction patterns of the composites show peaks related to the (111), (200) and (220) planes of face-centered cubic crystalline structures of metallic Ni, Co, and Cu with a trace amount of hexagonal cobalt (inserts in Fig. 1). In the diffraction pattern of the Cu/C sample, the weak peaks in the region of the most intense reflections of the copper oxides (CuO and Cu_2_O) were identified (inserts in Fig. 1). Semi-quantitative phase analysis by Match! showed the presence of Cu – 97 wt.%, Cu_2_O – 2 wt.%, and CuO – 1 wt.% in the Cu/C sample. Hence, only insignificant oxidation of the surface of Cu (0) particles occurred, according to the XRD data. The phase composition of the Ni/C and Co/C composites did not change.

The product showed no apparent reactivity towards oxygen if exposed to air at room temperature for a prolonged duration. Therefore, all samples were further investigated to determine the structure of the shell formed over the metal nanoparticles, which provides a high degree of protection from oxidation.

The composition of the nanocomposites was determined by X-ray fluorescence analysis (see Supplementary Table S3). The as-obtained results confirm the presence of the main phase of metallic nickel (84.8), cobalt (82.8), and copper (94.4 wt.%) with a trace amount of impurities of 0.033, 0.279, and 0.930 wt.% in Ni/C, Co/C, and Cu/C samples, respectively.

**Figure 2:**
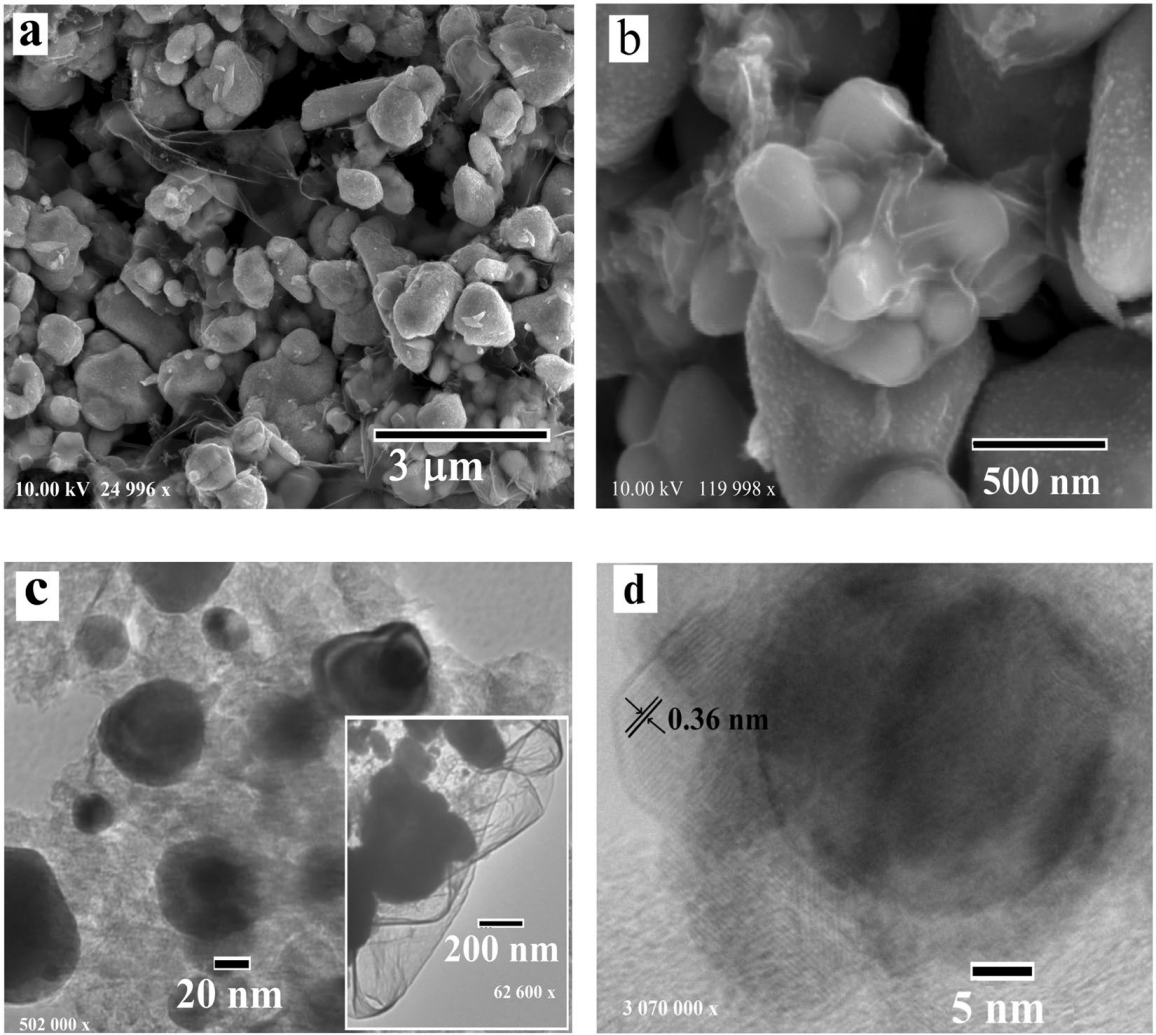
FIB-SEM (**a,b**) and TEM (**c,d**) images of the Cu/C nanocomposite after carbonization in an argon flow at 700 °C, demonstrating metal nanoparticles of 20–80 nm in diameter with crystalline graphitic shells with the thickness of 10–15 nm.

**Figure 3:**
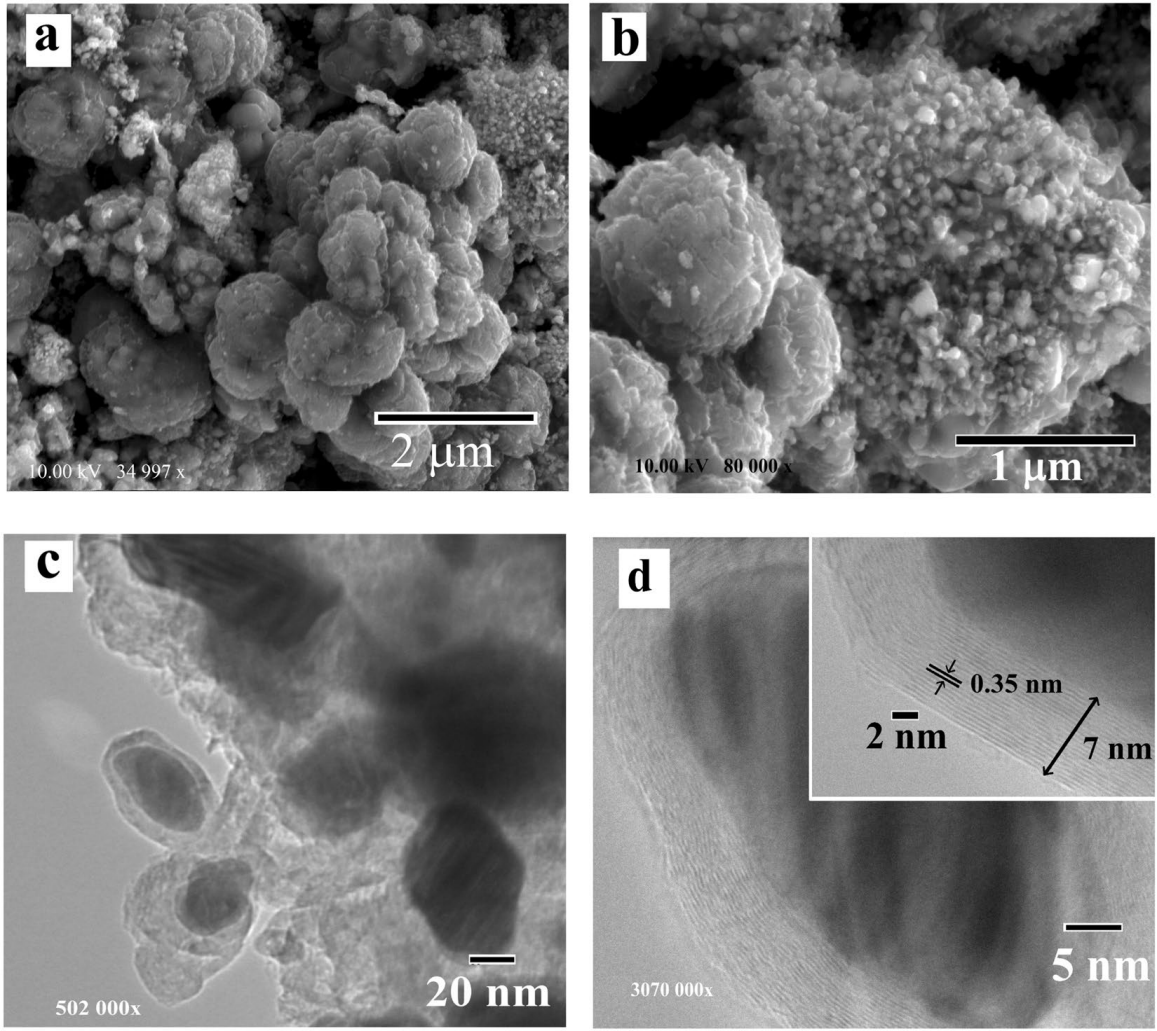
FIB-SEM (**a**,**b**) and TEM (**c,d**) images of the Co/C nanocomposite after carbonization in an argon flow at 700 °C, demonstrating metal nanoparticles of 10–20 nm in diameter with crystalline graphitic shells with the thickness of 7–10 nm.

**Figure 4:**
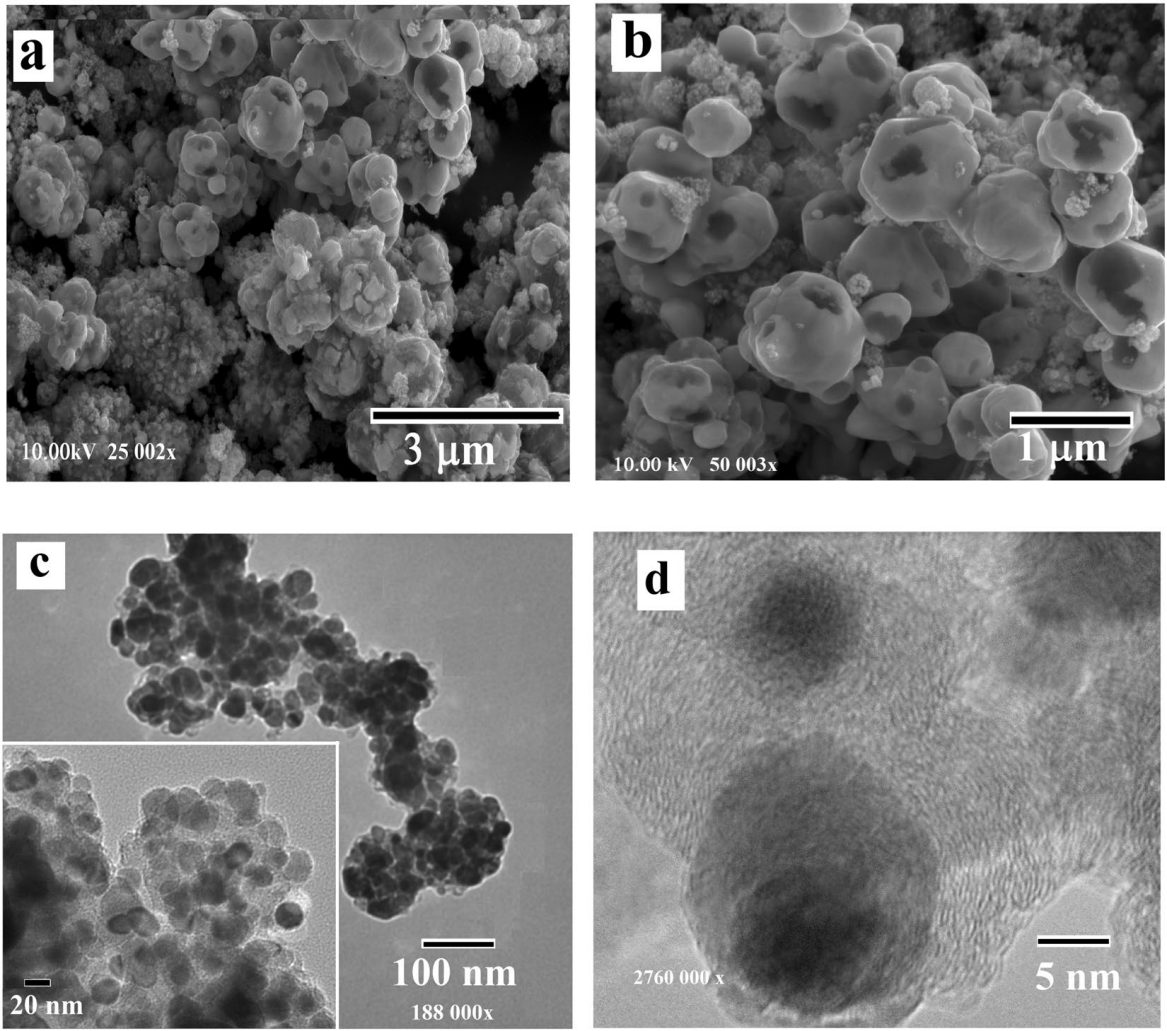
FIB-SEM (**a,b**) and TEM (**c,d**) images of the Ni/C nanocomposite after carbonization in an argon flow at 700 °C, demonstrating metal nanoparticles of 10–20 nm in diameter with crystalline graphitic shells with the thickness of 7–10 nm.

The morphology of the composites was characterized by focused ion beam-scanning electron microscopy (FIB-SEM) and transmission electron microscopy (TEM) methods. The FIB-SEM images (Figs. 2–4) show embedded and highly dispersed metal nanoparticles (bright light dots) within the carbon matrix (light grey pellets) as well as big metal clusters of irregular shape, located at the outer surfaces of the carbon nanocomposites, with diameters in the range of 50–500 nm. The concentrations of other carbon forms such as rods, fibers and tubes were negligible.

The TEM analysis demonstrated a core–shell nature of the nanocomposites with spherical-like metal nanoparticles of approximately 20–80 nm in size for Cu/C and 10–35 nm for Ni/C and Co/C, covered with 5–15 nm thick crystallized graphitic shells (Figs. 2–4). The average diameter of the metal nanoparticles examined by TEM corresponds well with the results based on the XRD data.

The formation of irregular particles, with brighter surfaces, of approximately 50–250 nm in size can be seen for the Cu/C composite (Fig. 2). Some distorted particles are composed of fused spheres. Furthermore, a thin, transparent, layered structure was observed for the Cu/C samples covering spherical particles of the nanocomposites (Fig. 2b,c).

The fragments at the surface of Co/C sample (Fig. 3a,b) have a similar sponge-like structure made of spheres with sharp fringes or broken microspheres up to 1 μm in diameter. Ni/C sample also contains spherical particles similar to the Co/C but with a smoother surface (Fig. 4a,b).

The lattice spacing of the shell is 0.35–0.36 nm for the different nanocomposites, which can be indexed to the (002) planes of graphite (Figs. 2–4). In the case of the Ni/C composite, the graphite shells are not uniform, but core nanoparticles are well embedded in the carbon matrix. TEM images of the nanocomposites confirm the particles agglomeration during carbonization (Fig. 4c,d). This may be due to the following reasons: inhomogeneous distribution of metal salts during polymerization, or redistribution of metal nanoparticles during heating caused by their migration and gathering effect.

Notably, with an equal molar ratio of metal-polymer in the initial mixtures, the carbon content in the composites after pyrolysis varies from 17.2 wt.% to 5.2 wt.% in the series Co > Ni > Cu (Table S1). Pyrolysis of mechanical mixtures of polymer and salts of Ni and Co can be accompanied by the formation of multilayer carbon shells on metal nanoparticles, as shown in the previous work^20^. During the carbonization process, the carbon species formed from the polymer, are decomposed catalytically and form carbon structures. The formation mechanism is different in all composites due to the different specific catalytic activities of the applied metals. This has a significant effect on the pyrolysis and hence on the composition of carbon, metal particles and composite structures. The formation of graphitic structures on Ni nanoparticles has been intensively studied because of its acceptability as a catalyst for high-quality graphite formation as well as nanotube growth. Copper has also been shown to catalyze the growth of such carbon allotropes as graphite, diamond, carbon nanotubes^33–37^. Recent results on growth of single layered graphene on the polycrystalline copper foils were presented by X. Li *et al*.^38^.

According to Mattevi *et al*. the ability to expel or precipitate carbon at the interfaces of metals to form sp^2^-bonded crystalline carbon from a solid solution depends on their carbon affinity^39^. Different solubility of carbon and affinity to carbon leads to the formation of stable or metastable carbides, or the absence of carbide phases. The affinity towards carbon decreases in the sequence Co, Ni, and Cu^40^. Thus, the best applicable catalysts for graphite formation are the metals with a low affinity towards carbon but with the ability to stabilize and maintain the growth of carbon on the surfaces through the formation of weak bonds. Furthermore, the mechanism of carbon deposition is defined by the solubility of carbon in the metal and the growth conditions, which eventually defines the morphology and thickness of graphite structures.

In accordance with the phase diagram of Ni and C, the solubility of carbon is low and therefore it will directly diffuse out of Ni^41^. The formation of a metastable carbide phase facilitates the formation of carbon out of Ni; the carbon, accordingly, mainly precipitates out of the Ni nanoparticles so the thickness of graphite at the nanoparticles boundaries is larger than within the nanoparticles, as outlined by Mattevi *et al*.^39^. Therefore, the number of graphite layers significantly varies along the entire surface of nickel nanoparticles in the synthesized Ni/C composite (Fig. 4d). Copper has the lowest affinity to carbon and does not form any carbide phases, it can form only weak bonds with carbon making copper a good catalyst for the formation of graphite (Fig. 2c,d). In general, the above-mentioned experimental results indicated that the as-obtained nanocomposites consist of outer carbon shells and inner core metal particles, where the outer shells can provide protection of the metal nanoparticles against oxidation.

**Figure 5:**
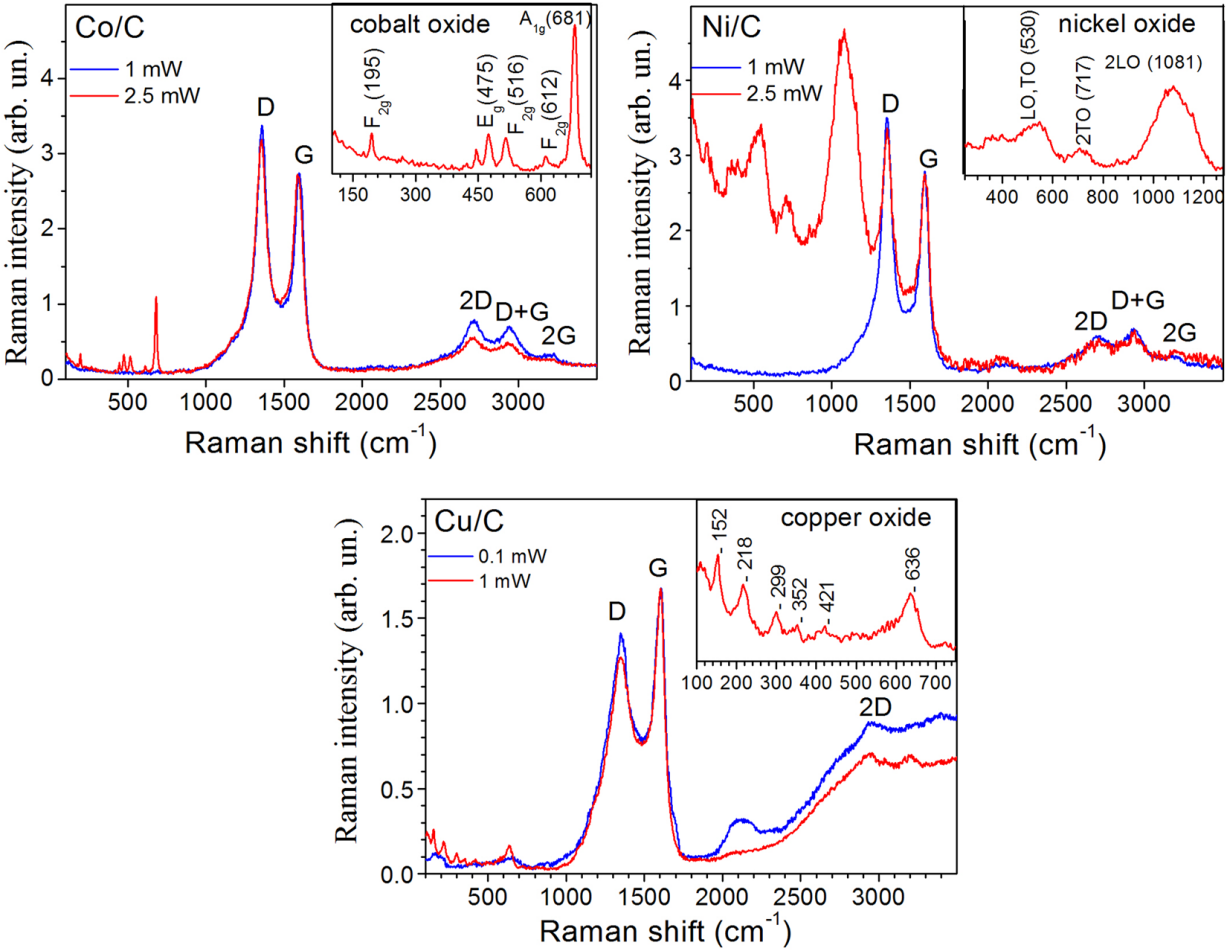
Raman spectra of Co/C, Ni/C, and Cu/C nanocomposites measured at laser wavelength λ_exc_ = 488 nm and varied excitation power. Spectra are normalized to the intensity of the G-band.

To further check the graphitic character of the composites, the materials were characterized by Raman spectroscopy. The Raman spectra were recorded in the range of 100–3500 cm^−1^, which is the most informative for carbon materials (Fig. 5). Raman spectroscopy is a non-destructive and powerful technique used to identify and characterize all the members of the carbon family^42,43^. Raman spectra measured at the lowest laser powers of 0.1 ÷ 1 mW are dominated by one-phonon peaks attributed to the G- (~1590 cm^−1^) and D-bands (~1350 cm^−1^) of disordered sp^2^ carbon. Less intense second-order peaks are attributed to 2D (~2710 cm^−1^), D + G (~2940 cm^−1^) and 2 G (~3200 cm^−1^) bands. In detail, the G-band refers to sp^2^ carbon atoms scattering due to vibrations of the E_2g_ mode, while the D-band represents internal defect-induced scattering. The shape of the Raman spectra indicates crystallization of graphitic structures with a low graphitization degree typical for nanographite as evidenced both by the rather high full-width of the G-band and the I_D_/I_G_ ratio (Table 1)^44^. The I_D_/I_G_ intensity ratio gives a measure of the structural disorder and makes it possible to estimate the average in-plane crystallite size (*L*_*a*_) of the sp^2^ domains according to the relation ${{I}}_{D}/{I}_{G}=0.0055{{L}_{a}}^{2}$^45,46^. As can be seen, the Cu/C nanocomposite shows the most disordered nanographite phase as evidenced by having both the highest full width of the G and D bands and the lowest estimated crystallite size *L*_*a*_.

**Table 1:** Position (ω) and full-width (Γ) of D- and G-bands, I_D_/I_G_ ratio and crystallite size (L_a_) estimated from the Raman spectra of the metal-carbon nanocomposites measured at a laser power of 0.1 mW.

Samples	D-band		G-band		I_D_/I_G_	L_a_, nm
	ω_D_, cm^−1^	Γ_D_, cm^−1^	ω_G_, cm^−1^	Γ_G_, cm^−1^		
Cu/C	1355.5	180.0	1598.6	95.5	0.79	1.20
Co/C	1356.9	91.1	1595.9	77.1	1.17	1.46
Ni/C	1353.3	84.8	1593.0	71.6	1.23	1.49

Therefore, Raman analysis demonstrated that nickel, in comparison to cobalt and copper, promotes the formation of a more ordered sp^2^ carbon structure. Enhanced formation of the more ordered nanographite phase leading to a higher thickness of carbon shell around the metal nanoparticles, therefore, appears to be the reason for the increased carbon content in the composites obtained particularly using Co and Ni.

Increase of exciting laser power up to 1–2.5 mW results in the appearance of a series of additional low-frequency Raman bands, that can be related to laser-induced oxidation of the metal nanoparticles with a resultant formation of cobalt oxide, copper oxide and nickel oxide correspondingly for the Co/C, Cu/C and Ni/C nanocomposites. The most prominent peaks appeared in the Raman spectrum of the Co/C at 2.5 mW can be assigned to E_g_(475 сm), F_2g_(195, 516 and 612 cm^−1^) and A_1g_ (681 cm^−1^) modes of the Co_3_O_4_ crystalline phase, whereas the low-frequency shift of about 7.5, 0.5, 5.4, 6.0 and 10 cm^−1^ for each of the peaks respectively, as compared to bulk Co_3_O_4_ and asymmetric broadening can be attributed to phonon confinement effects^47–49^. For the Ni/C at 2.5 mW, the broad Raman peak at ~546 cm^−1^ is assigned to the first-order scattering on LO and TO phonons of NiO, whereas the peaks at ~717 and 1081 cm^−1^ are related to two-phonon 2TO and 2LO scattering, correspondingly^50^. The absence of the two-phonon TO + LO band expected at ~906 cm^−1^ for bulk NiO and rather high relative intensity of the one-phonon band is related to the inherent disorder in nanosized NiO due to defects or surface effects^51^. The set of Raman peaks for the Cu/C at 1 mW found at 152, 218, 299, 352, 421 and 636 cm^−1^ correspond to formation of mixed CuO and Cu_2_O oxides^52–56^.

The least resistivity to laser-induced oxidation is shown by the Cu/C composite, where the formation of copper oxide is already observed at just 1 mW of laser power. The thicker carbon layers of the Co/C and Ni/C provide greater protection, and hence greater laser power in order to observe oxidation. The presence of a surface oxide layer of the metal nanoparticles could also prevent their oxidation by atmospheric oxygen in the absence of active chemical reagents, leading to high nanoparticle stability over time^57^.

To identify the surface states of the prepared samples the X-ray photoelectron spectra were recorded (see Supplementary Fig. S3). The characteristic peaks in the photoelectron spectra for Ni 2p and Cu 2p confirmed the presence of metallic Ni(0) and Co(0), as well as Co_2_O_3_ and CoO forms in the Co/C sample, and NiO phase in the Ni/C composite. The Cu 2p spectra show the formation of a hybrid phase of zero-valent copper, CuO and Cu_2_O. The obtained data are in accordance with the XRD result.

It is worth noting that the powders of the composites were hydrothermally treated after carbonization to study the hydrolytic stability of the composites^32^. The experiment showed that the resistance to hydrolysis decreases in the series Ni/C > Cu/C ≈ Co/C. There was slight oxidation of metal phase with the formation of copper oxide Cu_2_O and cobalt hydroxide Co(OH)_2_ in the Cu/C and Co/C composites, respectively (see Supplementary Fig. S4). The content of these phases was about 5 wt.% towards metallic phases and with a crystallite size of ~10–12 nm.

## Materials and Methods

Copper acetate monohydrate Cu(CH_3_CHOO)_2_·H_2_O (GOST 5852-79), cobalt acetate tetrahydrate Co(CH_3_CHOO)_2_·4H_2_O (GOST 5861-79), nickel acetate tetrahydrate Ni(CH_3_CHOO)_2_ 4H_2_O (TU 6-09-3848-87) and polyethylene glycol 1500 (TU 2483-008-71150986-2006) were used for the synthesis of samples. All chemical reagents used in this study were used without further purification.

The synthesis of metal-carbon nanocomposites was carried out in two stages. In the first stage, the metal acetate was ground with a polymer in a ball mill with a porcelain drum. In the second, the obtained mixture was pyrolyzed in a vertical stainless-steel reactor in an argon flow at 700 °C for 2 h. The samples were discharged after cooling to room temperature in the reactor in a stream of argon. The molar ratio of the metal acetate and the macromolecule unit of polyethylene glycol for all samples was 1:4.

The morphology of the metal-carbon nanocomposites was analyzed by the xT Nova Nanolab 600 FIB (FIB-SEM) that combines both an electron and a Gillum ion beam simultaneously. This dual beam system is capable of providing high-quality image resolution down to the sub-nanometre range (in comparison to a standard scanning electron microscopy).

To get higher magnification and determine the layered structure of the carbonaceous part of the nanocomposites, transmission electron microscopy (TEM) was employed. TEM samples of nanometer order were dispersed in isopropanol and subsequently drop-cast onto holey carbon films on 200 mesh Cu grids purchased from Agar Scientific (#AGS147H).

Powder X-ray diffraction (XRD) patterns were recorded using DRON UM1 and DRON-4-07 diffractometers (Burevestnik, St.-Petersburg, Russia) with monochromatic CoK_α_ and filtered CuK_α_ radiation for Ni, Co- and Cu- containing samples, respectively, at 2θ  = 10–80°. The average sizes of crystallites were calculated from the full width at half the maximum of the corresponding XRD peaks, using the Scherrer equation^58^. Semi-quantitative phase analysis was made using Match!^59^.

To analyze the textural characteristics, low-temperature (77.4 K) nitrogen adsorption-desorption isotherms were recorded using Sorptometer KELVIN 1042 of COSTECH Instruments. The samples were pre-degassed in a stream of helium at 120 °C for 2 hours. The volume of the adsorbed gas was determined at the time of quasi-equilibrium in the gas low controlled by a thermal conductivity detector (the accuracy of the measurement was 3%). The specific surface area of the samples was determined by the BET method^60^. The pore diameters were calculated using the desorption branch of the isotherm by the modified BJH method.

The XPS measurements were performed in a factory XPS spectrometer JEOL “JSPM-4610” in operating vacuum 10^−7^ Pa with monochromatic 1486.6 eV AlK_α_ radiation. The energy resolution of the spectrometer was 0.9 eV (FWHM). Calibration of the bonds energy was carried out upon 4*f*-line with the energy of 87.5 eV for Au with the purity of 99.999%^61^.

X-ray fluorescence method (XRF) was used to study the chemical composition of the composites. Photoelectron spectra were recorded with EXPERT Mobile (INAM, Ukraine) electron spectrometer equipped with SDD-detectors with a typical resolution of 145 eV for MnK_α_ X-ray source. The range of the measured element contents is 0.005–100%. The determination of elements is from magnesium 12Mg to uranium 92U (optional 11Na) simultaneously in a single measurement^62^.

The structure of the nanocomposites were characterized using micro‐Raman spectroscopy at appropriate experimental conditions^63^. The measurements were performed at room temperature in backscattering configuration using a triple Raman spectrometer T‐64000 Horiba Jobin‐Yvon, equipped with electrically cooled charge‐coupled device (CCD) detector and Olympus BX41 microscope. The Ar‐Kr ion laser line with wavelength of 488 nm was used for excitation. The excitation radiation was focused on the sample surface through a × 50/NA 0.75 optical objective, giving a laser spot diameter of about 1 μm. The laser power incident on the sample surface was varied in the range of 0.1−2.5 mW.

## Conclusions

Metal-carbon nanocomposites, based on copper, nickel, and cobalt, were successfully obtained using mechanochemical and pyrolytic methods of synthesis. The essence of the method consists of the interaction of the polymer and metal compounds as a result of the joint grinding of metal-containing salts and the polymer phases. Next, the carbonization of the obtained composition is carried out, as a result of which the polymer is carbonized and the metal compounds are completely reduced. The resulting carbon material is nanostructured in the form of formations of various shapes and sizes. A metal-carbon nanocomposite is a metal nanoparticle stabilized by outer carbon structures.

The as-prepared materials have a core-shell structure with average sizes of metal nanocrystallites of 50, 18, and 20 nm, and carbon contents of 5.6, 15.2, and 17.2 wt.%, respectively. The formation of carbon structures in the samples has been observed, which formed a stable long-term protective shield against air and moisture impact. The carbon layer consisted of sp^2^-hybridized nanographite stacks with a low degree of crystalline order and a spherical shape. It was shown that Ni and Co are beneficial for the formation of the graphite phase during the carbonization of organic substances due to the enhanced formation of more ordered nanographite phase. XPS analysis confirmed that the surface states of the metal-carbon nanocomposites consists of the different surface intermediate amorphous layers. The obtained composites show good resistance towards oxygen from air over time due to the presence of the carbon shell and the surface oxide layer of the metal nanoparticles.

The proposed method of mechanochemical treatment with subsequent pyrolysis allows one to synthesize a wide range of compositions, sizes, and morphologies of metal-carbon nanocomposites, to regulate their structure, and to expand the scope of such materials. By controlling the size and shape of nanostructures, by changing the amount of the metal-containing phase and polymer, it is possible to impart new properties to the materials, which sharply distinguish them from ordinary compositions. The prepared metal-carbon nanocomposites are promising for use in the fabrication of electronic, electrochemical, and sensor devices as alternative sensor material for temperature and pressure sensing, as well as catalysts.

## Supplementary information

Supplementary Information

## Data Availability

The authors declare that all relevant data are included in the paper and in Supplementary Information files.
