# Supplementary material for: Mechanochemical synthesis of carbon-stabilized Cu/C, Co/C and Ni/C nanocomposites with prolonged resistance to oxidation
Source: Sci Rep. 2019 Nov 22;9:17435. doi: 10.1038/s41598-019-54007-2 (PMC6874553; doi:10.1038/s41598-019-54007-2)
Supplement: Supplementary file 1 — Supplementary Information [file 41598_2019_54007_MOESM1_ESM.pdf]

Supplementary material

Mechanochemical synthesis of carbon-stabilized Cu/C, Co/C and Ni/C nanocomposites with prolonged resistance to oxidation

Mariia Galaburda[1], Evgeniya Kovalska[2], Benjamin T. Hogan[2], Anna Baldycheva[2], Andrii Nikolenko[3], Galina I. Dovbeshko[4], Olena I. Oranska[1] & Viktor M. Bogatyrov[1]

[1]Oxide Nanocomposites Laboratory, Chuiko Institute of Surface Chemistry of NAS of Ukraine, 17 General Naumov Str. Kyiv, 03164, Ukraine
[2]Department of Engineering and Centre for Graphene Science, College of Engineering, Mathematics and Physical Sciences, University of Exeter, Exeter, EX4 4QF, United Kingdom
[3] Optical Submicron Spectroscopy Laboratory, Institute of Semiconductor Physics of NAS of Ukraine, 45 Nauky Ave. Kyiv, 02000, Ukraine
[4] Department of Physics of Biological Systems, Institute of Physics of NAS of Ukraine, 46 Nauky Ave. Kyiv, 02000, Ukraine
* Correspondence: mariia.galaburda@gmail.com. (M.G.) Tel.: +38-044-422-9672.

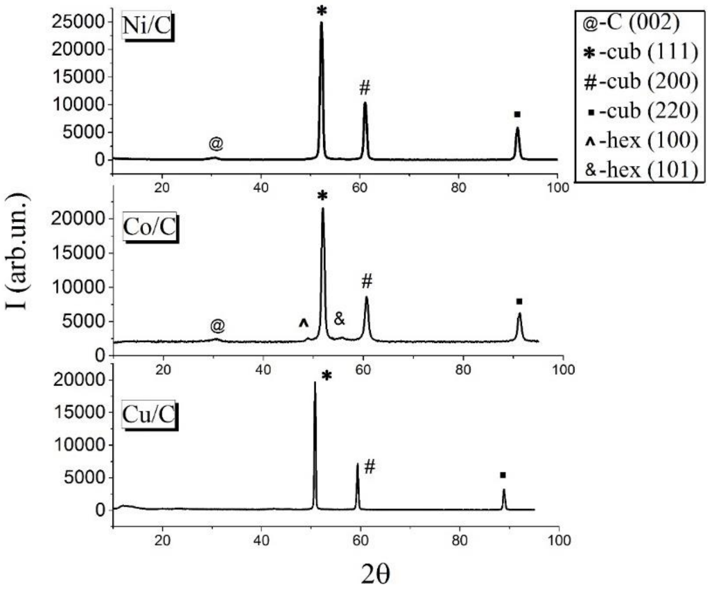

Figure S1. XRD patterns of Ni/C, Co/C, and Cu/C nanocomposites, obtained via pyrolysis under argon flow at 700 °C, D_{cr} of Co/C and Ni/C is 18-20 nm, and 50 nm for Cu/C.

Table S1. Characteristics of the Ni/C, Co/C, and Cu/C nanocomposites.

Samples	S_{BET}, m^2/g	Metal content, wt.%.	Carbon content, wt.%.	Metal D_{cr}, nm	Graphite D_{cr}, nm
Cu/C	39	94,4	5.6	50	-
Co/C	41	82,8	17.2	20	5-7
Ni/C	28	84,8	15.2	18	5-7

To analyze the textural characteristics, low-temperature (77.4 K) nitrogen adsorption–desorption isotherms were recorded. The adsorption/desorption isotherms (type IV of IUPAC classification) show that they represent mesoporous composites giving low specific surface area (TableS1, S_{BET}) and small pore volume (V_p) with broad PSD (Fig.1S).

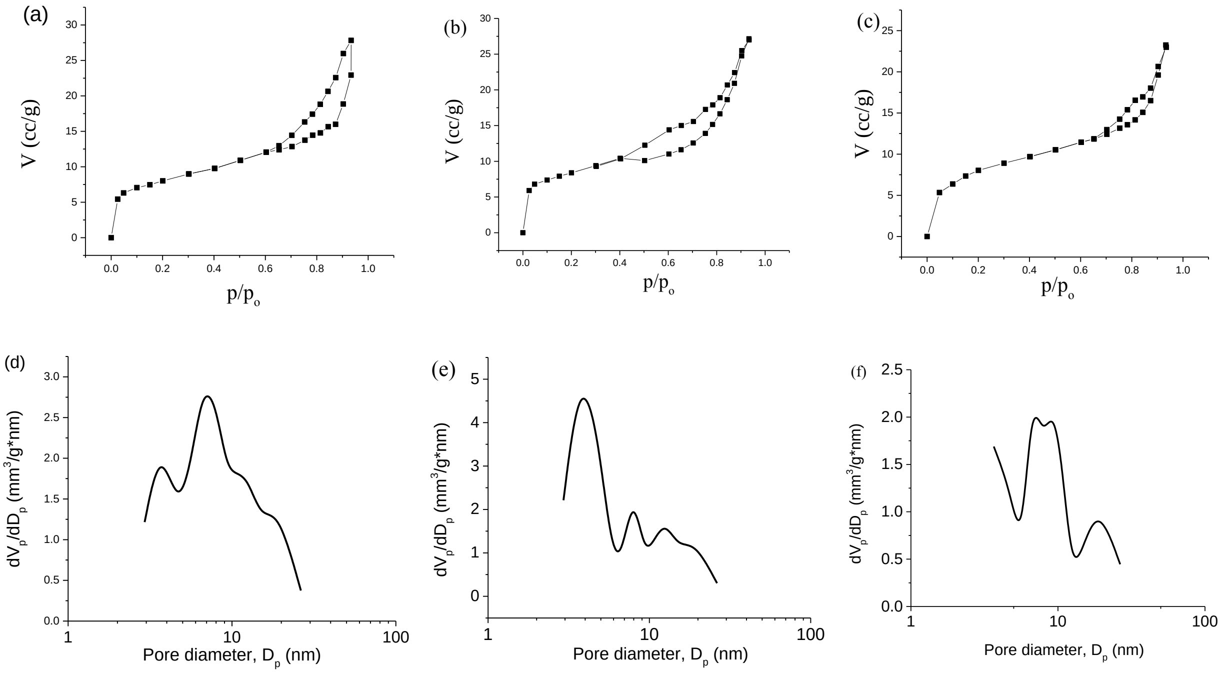

Figure S2. Nitrogen adsorption-desorption isotherms (a,b,c) and pore size distributions (d,e,f) of Ni/C (a,d), Co/C (b,e), and Cu/C (c,f) nanocomposites.

Table S2. Structural characteristics of metal-carbon composites.

	Ni/C	Co/C	Cu/C
BET Surface Area, m^2/g	28	8.94	29
Micropore Volume, mm^3/g	2.1	2.4	0.1
Total pore volume, mm^3/g	43.0	42.0	36.0

Table S3. The impurity contents in the nanocomposites according to the X-ray fluorescence analysis.

Sample	Metal content, wt.%	Impurity content, wt.%	$\Sigma_{impurity}$, %
Cu/C	94,4	Co – 0.611; Si – 0.047; In – 0.084; S – 0.188	0.930
Co/C	82,8	Fe – 0.015; Ni – 0.249; S - 0.015;	0.279
Ni/C	84,8	Fe – 0.025; Si – 0.008	0.033

The XPS spectra in the binding energy range of 0-1000 eV were obtained to identify the surface states of the prepared samples. XPS measurements for the nanocomposites were carried out recording the $O1_s$, and metal 2p core-level spectra.

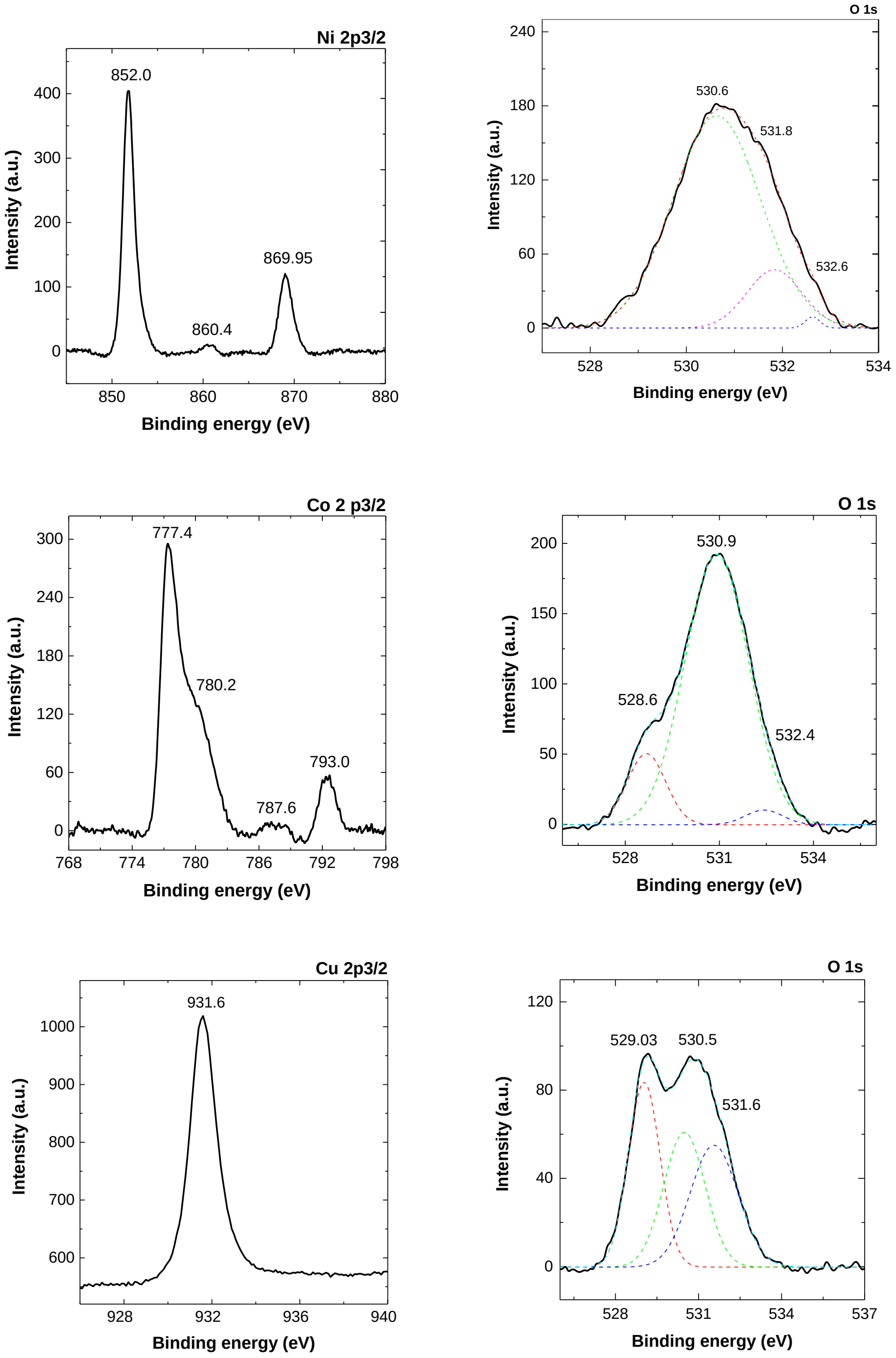

Figure S3. XPS spectra of Ni 2p, Co 2p, and Cu 2p, and O 1s regions from the Ni/C, Co/C and Cu/C composites.

The Ni $2p_{3/2}$ region has two components at 852.0 and 860.40 eV. The components with binding energy (BE) values of 852.0 and 869.95 eV are characteristic of metallic nickel [1,2]. The second component of the Ni $2p_{3/2}$ region at 860.40 eV correspondes to a satellite of the main peak characteristic of a Ni(II)–O bond [3, 4].

The Co $2p_{3/2}$ region consisted of peaks at 777.4 and 793 eV which correspondes to metallic Co and a satellite feature detected at 780 -787 eV suggested a Co_2O_3 and CoO composition. The small shift of the main picks at higher BE can be assigned to oxygen atoms bound to the metal.

High-resolution XPS spectra reveal one sharp Cu $2p_{3/2}$ peak at 931.6 eV can be assigned to the Cu according to the position of the LMM peaks in the Auger spectra. 0.9 eV shift to the lower binding energy can be caused by the presence of oxide form that is defined by the composite nature. The formation of the CuO and Cu_2O composition is confirmed by the appearance of two peacks at BE of 529 and 530.5 eV.

O 1s peaks tend to be broad, with multiple overlapping components. Thus, the band in the O 1s region was deconvoluted into three peaks. Two of them are attributed to the oxide phases of metals (with BE of 528-530.5 eV). The component located at ≈ 532.4 eV is usually attributed to the presence of loosely bound oxygen on the oxide surface, such as $-CO_3$, adsorbed H_2O, and adsorbed O_2, The component with BE of 531.6 eV can be referred to C=O (carbonyl and carboxyl).

Comparison of diffractograms of the initial and hydrothermally treated metal-carbon composites (Figures S1 and S4) showed the identity of the phase composition and the average crystallite size of the metal nanoparticles of Ni-containing composites and revealed differences in these characteristics in Cu-, Co-containing samples. Cu_2O (JCPDS № 74-1230) was detected in the Cu/C composite and $Cu(OH)_2$ (JCPDS № 74-1057) After hydrothermal treatment, Co_2O copper oxide (JCPDS No. 74-1230), and $Co(OH)_2$ (JCPDS No. 74-1057) in the Co/C sample with the content of these phases of about 5 wt. % relative to Cu and Co. The crystallite size was ~ 10-12 nm. At the same time, the size of Cu crystallites increased to 57 nm, and the size of Co crystallites decreased to 17 nm.

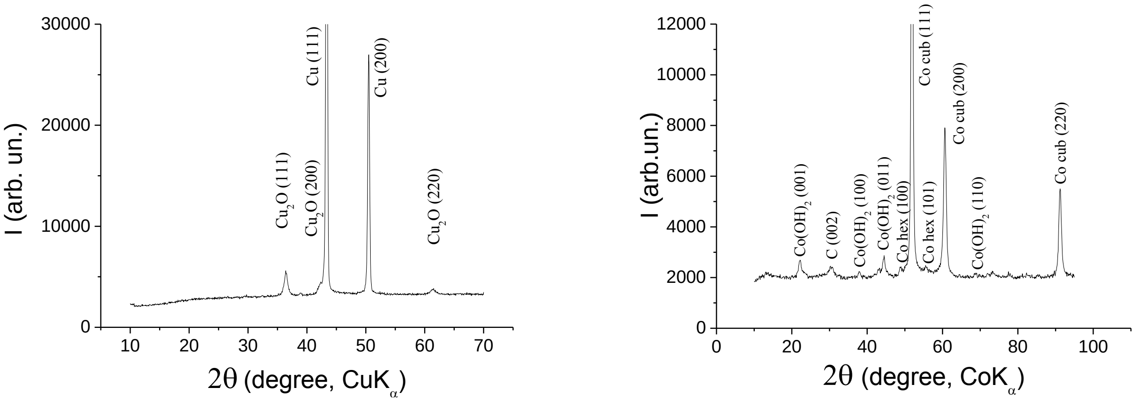

a ***b***

Figure S4. XRD patterns of Cu/C (a) and Co/C (b) nanocomposites after hydrothermal treatment.

References

1. http://srdata.nist.gov/xps.
2. https://srdata.nist.gov/xps/XPSDetailPage.aspx?AllDataNo=111408
3. Moreno-Castilla, C., Maldonado-Hodar, F.J., Perez-Cadenas, A.F. Physicochemical surface properties of Fe, Co, Ni, and Cu-doped monolithic organic aerogels. *Langmuir* **19** 5650–5655 (2003).
4. https://srdata.nist.gov/xps/XPSDetailPage.aspx?AllDataNo=112464#General.htm
